# Etiology in Children Presented With Chronic Liver Disease in a Tertiary Care Hospital

**DOI:** 10.7759/cureus.25570

**Published:** 2022-06-01

**Authors:** Ayesha Sardar, Arit Parkash, Ayesha A Merchant, Bushra Qamar, Faryal Ayub, Shamama Zehravi

**Affiliations:** 1 Pediatric Medicine, National Institute of Child Health, Karachi, PAK

**Keywords:** hepatitis b (hbv), wilson disease, glycogen storage disorder, idiopathic, chronic liver disease (cld)

## Abstract

Background

Chronic liver disease (CLD) is one of the most significant causes of morbidity and mortality among the pediatric age group. The identification of the etiology of the disease is of utmost importance for the effective management of the disease.

Objective

To determine the various causes of CLD in pediatric patients attending a large public sector pediatric hospital.

Patients and methods

A prospective cross-sectional study was conducted at the National Institute of Child Health, Karachi, Pakistan from August 1, 2021, to February 28, 2022. All patients below 16 years of age of either gender with more than three months of symptoms duration on admission were enrolled. The diagnosis was labeled after the standard reference ranges for the pediatric age group.

Results

Of 136 patients, the mean age was 4.42 ± 3.92 years. More than half of the patients were males (76, 55.9%). Hepatitis B (31, 22.8%), idiopathic (23, 16.9%), glycogen storage disorder (GSD) (21, 15.4%), and Wilson disease (14, 10.3%) were the most common cause of CLD. A significant association of hepatitis was observed with age (p-value < 0.001), residence (p-value = 0.048), symptomatic (p-value < 0.001), total bilirubin level (p-value = 0.003), direct bilirubin level (p-value = 0.002), and albumin level (p-value = 0.003). Whereas a significant association of GSD was observed with age (p-value = 0.001), residence (p-value < 0.001), and serum glutamic pyruvic transaminase (SGPT) level (p-value = 0.033).

Conclusion

In our cohort, hepatitis B, idiopathic, GSD, and Wilson disease were the most common causes of CLD in pediatric patients. Moreover, age, residence, symptomatic, total bilirubin level, direct bilirubin level, SGPT, and albumin level were the important predictor variables.

## Introduction

Chronic liver disease (CLD) is defined as progressive destruction and regeneration of liver parenchyma, which has been presented for at least six months, leading to fibrosis and cirrhosis [1,2]. CLD is one of the most significant causes of morbidity and mortality among the pediatric age group [3].

A number of etiologic factors have been identified in children with CLD including a broad spectrum of disorders, which is different from adults, such as infections, developmental abnormalities (such as biliary atresia can lead to biliary cirrhosis if left untreated), and metabolic and neoplastic diseases leading to hepatic failure [4,5].

The prognosis and treatment of CLD are influenced by the etiology of the disease, thus it is critical to figure out the triggering factor. It necessitates a clinicopathological evaluation that involves a thorough history, examination, and a number of diagnostic tests. Although advancements have been made in the diagnostic field, to detect and categorize liver damage non-invasively, a biopsy of the liver is the gold standard for liver disease diagnostics [6,7].

The rationale of this study is to determine the different causes of chronic liver failure among children presented to cater to patients from different parts of the country so that the etiological spectrum in the Pakistani population could be known, which might help in taking the relevant preventive measures accordingly. Keeping in view the late presentation of the cases in our part of the world, the identification of etiological factors will aid in the early recognition of the disease.

## Materials and methods

This prospective cross-sectional study was conducted at the National Institute of Child Health, Karachi, Pakistan from August 1, 2021, to February 28, 2022. Ethical approval was obtained from the institution prior to conducting the study (IERB-49/2019). Furthermore, parents/guardians of all study participants provided informed permission after being informed of the study.

All children below 16 years of age of either gender having symptoms duration of more than three months on admission to the hospital were included in the study. Whereas thalassemia patients with recurrent transfusions and patients with acute liver failure were excluded.

The sample size was calculated with the help of OpenEpi, version 3, an open-source calculator. The calculated sample size was 136 cases with a 95% confidence level, a margin of error of 5%, and taking the expected percentage of hepatitis B virus at 9.8% [8] as a cause of CLD in children.

Related investigations and imagining studies were carried out. Laboratory investigations such as complete blood count, liver function test, and albumin were recorded. Ultrasound (US) of the abdomen was performed in all patients. Unnecessary investigations were avoided and those with unknown causes were labeled as “idiopathic” after ruling out common causes of CLD. Furthermore, the diagnosis was labeled after the standard reference ranges for the pediatric age group met. This information, as well as other demographic and clinical factors, were recorded.

SPSS version 24 (IBM Corp., Armonk, NY) was used for statistical analysis. Mean ± standard deviation (SD) was computed for quantitative variables like age, duration of symptoms, hemoglobin level, total leucocyte count, platelet count, total bilirubin level, direct bilirubin level, serum glutamic pyruvic transaminase (SGPT), alkaline phosphate (ALP), and albumin level. Frequency and percentages were calculated for qualitative variables like gender, residence, religion, splenomegaly, hepatomegaly, and etiological spectrum of CLD. The mean difference of quantitative variables with respect to common causes of CLD was explored using an independent t-test. Whereas the association of these outcome variables with independent qualitative factors was explored using the chi-square/Fisher's exact test. A p-value of ≤ 0.05 was considered significant.

## Results

A total of 136 patients were enrolled. The mean age of the patients was 4.42 ± 3.92 years. There were 59 (43.4%) patients younger than or equal to two years of age and 77 (56.6%) patients were older than two years. More than half of the patients were males (76, 55.9%). The residence of 56 (41.2%) patients was rural. The mean duration of symptoms was 4.26 ± 4.72 months (Table 1).

**Table 1:** Baseline characteristics of the patients (n = 136) * Twenty-three patients were asymptomatic.

	N	%
Age, years (mean ± SD)	4.42 ± 3.92	
≤2	59	43.4
>2	77	56.6
Gender		
Male	76	55.9
Female	60	44.1
Residence		
Rural	56	41.2
Urban	80	58.8
Religion		
Islam	128	94.1
Other	8	5.9
Duration of symptoms, months (mean ± SD) (n = 113)*	4.26 ± 4.72	
≤5	83	73.5
>5	30	26.5

The laboratory characteristics showed that the mean hemoglobin level was 10.02 ± 2.07 g/dl, total leucocyte count was 9.12 ± 5.32 per liter, platelet count was 293.27 ± 148.10 per mcL, total bilirubin level was 4.83 ± 4.32 mg/dL, direct bilirubin level was 2.75 ± 2.87 mg/dL, SGPT was 106.66 ± 91.68 units per liter, ALP was 106.66 ± 91.68 units per liter, while the mean albumin level was found to be 344.17 ± 238.28 units per liter. Splenomegaly was observed in 10 (7.4%) and hepatomegaly in 62 (45.6%) patients (Table 2).

**Table 2:** Clinical and laboratory characteristics of the patients (n = 136) ALP: alkaline phosphatase; dl: deciliter; g: gram; mcL: microliter; mg: milligram; SGPT: serum glutamic pyruvic transaminase.

Laboratory characteristics	Mean ± SD
Hemoglobin, g/dl	10.02 ± 2.07
Total leucocyte count, per liter	9.12 ± 5.32
Platelet count, per mcL	293.27 ± 148.10
Total bilirubin, mg/dL	4.83 ± 4.32
Direct bilirubin, mg/dL	2.75 ± 2.87
SGPT, units per liter	106.66 ± 91.68
ALP, units per liter	344.17 ± 238.28
Albumin	3.39 ± 0.51
Clinical characteristics	n (%)
Splenomegaly	10 (7.4)
Hepatomegaly	62 (45.6)

The etiological spectrum of CLD showed that hepatitis B was the most common cause reported in 31 (22.8%) patients, followed by idiopathic cause in 23 (16.9%), glycogen storage disorder (GSD) in 21 (15.4%), Wilson disease in 14 (10.3%), biliary atresia in 10 (7.4%), autoimmune and progressive familial intrahepatic cholestasis in seven (5.1%) each, tyrosinemia type-I in six (4.4%), galactosemia and Budd-Chiari syndrome in four (2.9%) each, neonatal hepatitis in three (2.2%), Niemann-Pick disease and choledochal cyst in two (1.5%) each, while citrin deficiency and hypothyroidism were observed in one (0.7%) patient each (Table 3).

**Table 3:** Etiological spectrum of chronic liver disease with respect to baseline characteristics (n = 136) All data are presented as n (%). GSD: glycogen storage disorder; PFIC: progressive familial intrahepatic cholestasis.

	Total (n = 136)	Age ≤ 2 years (n = 59)	Age > 2 years (n = 77)	Male (n = 76)	Female (n = 60)	Symptomatic (n = 113)	Asymptomatic (n = 23)
Hepatitis B	31 (22.8)	2 (3.4)	29 (37.7)	20 (26.3)	11 (18.4)	14 (12.3)	17 (73.9)
Idiopathic	23 (16.9)	9 (15.3)	14 (18.2)	12 (15.8)	11 (18.3)	22 (19.5)	1 (4.3)
GSD	21 (15.4)	16 (27.1)	5 (6.5)	10 (13.2)	11 (18.3)	20 (17.7)	1 (4.3)
Wilson disease	14 (10.3)	4 (6.8)	10 (13.0)	3 (3.9)	11 (18.3)	14 (12.4)	0 (0)
Biliary atresia	10 (7.4)	9 (15.3)	1 (1.3)	5 (6.6)	5 (8.3)	9 (8.0)	1 (4.3)
Autoimmune	7 (5.1)	0 (0)	7 (9.1)	5 (6.6)	2 (3.3)	7 (6.2)	0 (0)
PFIC	7 (5.1)	6 (10.2)	1 (1.3)	4 (5.3)	3 (5.0)	7 (6.2)	0 (0)
Tyrosinemia type-I	6 (4.4)	4 (6.8)	2 (2.6)	6 (7.9)	0 (0)	5 (4.4)	1 (4.3)
Galactosemia	4 (2.9)	4 (6.8)	0 (0)	3 (3.9)	1 (1.7)	4 (3.6)	0 (0)
Budd-Chiari syndrome	4 (2.9)	0 (0)	4 (5.2)	3 (3.9)	1 (1.7)	1 (4.3)	0 (0)
Neonatal hepatitis	3 (2.2)	3 (5.1)	0 (0)	1 (1.3)	2 (3.3)	3 (2.7)	0 (0)
Niemann-Pick disease	2 1.5)	0 (0)	2 (2.6)	2 (2.6)	0 (0)	2 (1.8)	0 (0)
Choledochal cyst	2 (1.5)	0 (0)	2 (2.6)	1 (1.3)	1 (1.7)	2 (1.8)	0 (0)
Citrin deficiency	1 (0.7)	1 (1.7)	0 (0)	0 (0)	1 (1.7)	0 (0)	1 (4.3)
Hypothyroidism	1 (0.7)	1 (1.7)	0 (00	1 (1.3)	0 (0)	1 (0.9)	0 (0)

A significant association of hepatitis was observed with age (p-value < 0.001), residence (p-value = 0.048), symptomatic (p-value < 0.001), total bilirubin level (p-value = 0.003), direct bilirubin level (p-value = 0.002), and albumin level (p-value = 0.003). Whereas a significant association of GSD was observed with age (p-value = 0.001), residence (p-value < 0.001), and SGPT level (p-value = 0.033) (Table 4).

**Table 4:** Comparison of most common CLD causes with baseline characteristics (n = 136) All data are presented as n (%). Ϯ Chi-square test/Fisher's exact test applied. ∫ Independent t-test applied. P-value ≤ 0.05 is considered significant. * p-value ≤ 0.05. CLD: chronic liver disease; GSD: glycogen storage disorder; TLC: total leucocyte count; SGPT: serum glutamic pyruvic transaminase; ALP: alkaline phosphatase.

	Hepatitis			Idiopathic			GSD		
Variables	Yes (n = 31)	No (n = 105)	P-value^ Ϯ^	Yes (n = 23)	No (n = 113)	P-value^ Ϯ^	Yes (n = 21)	No (n = 115)	P-value^ Ϯ^
Age, years									
≤2	2 (3.4)	57 (96.6)	<0.001^Ϯ^*	9 (15.3)	50 (84.7)	0.652	16 (27.1)	43 (72.9)	0.001*
>2	29 (37.7)	48 (62.3)		14 (18.2)	63 (81.8)		5 (6.5)	72 (93.5)	
Gender									
Male	20 (26.3)	56 (73.7)	0.271^Ϯ^	12 (15.8)	64 (84.2)	0.694	10 (13.2)	66 (86.8)	0.407
Female	11 (18.3)	49 (81.7)		11 (18.3)	49 (81.7)		11 (18.3)	49 (81.7)	
Residence									
Rural	8 (14.3)	48 (85.7)	0.048^Ϯ^	7 (12.5)	49 (87.5)	0.251	16 (28.6)	40 (71.4)	<0.001*
Urban	23 (28.8)	57 (71.3)		16 (20.0)	64 (80.0)		5 (6.3)	75 (93.8)	
Religion									
Islam	30 (23.4)	98 (76.6)	0.682^‡^	20 (15.6)	108 (84.4)	0.133	21 (16.4)	107 (83.6)	0.609
Other	1 (12.5)	7 (87.5)		3 (37.5)	5 (62.5)		0 (0)	8 (100)	
Symptomatic									
Yes	14 (12.4)	99 (87.6)	<0.001^Ϯ^*	22 (19.5)	91 (80.5)	0.124	20 (17.7)	93 (82.3)	0.126
No	17 (73.9)	6 (26.1)		1 (4.3)	22 (95.7)		1 (4.3)	22 (95.7)	
	Mean ± SD	Mean ± SD	P-value^∫^	Mean ± SD	Mean ± SD	P-value^∫^	Mean ± SD	Mean ± SD	P-value^∫^
Hemoglobin, g/dl	10.31 ± 2.42	9.94 ± 1.96	0.383	9.54 ± 1.88	10.12 ± 2.10	0.223	10.13 ± 1.21	10.01 ± 2.19	0.804
TLC, per liter	8.03 ± 4.35	9.44 ± 5.56	0.194	8.26 ± 4.18	9.29 ± 5.53	0.394	10.69 ± 4.88	8.83 ± 5.37	0.144
Platelet count, per mcL	285.0 ± 97.08	295.7 ± 160.3	0.725	290.69 ± 195.74	293.80 ± 137.51	0.927	359.95 ± 146.15	281.10 ± 145.80	0.024
Total bilirubin, mg/dL	2.83 ± 3.55	5.42 ± 4.37	0.003*	3.82 ± 3.46	5.04 ± 4.46	0.221	4.62 ± 4.05	4.87 ± 4.39	0.809
Direct bilirubin, mg/dL	1.29 ± 2.25	3.19 ± 2.91	0.002*	2.27 ± 2.45	2.85 ± 2.95	0.382	2.46 ± 2.56	2.81 ± 2.94	0.611
SGPT, units per liter	104.2 ± 98.25	107.3 ± 90.14	0.870	96.21 ± 83.27	108.79 ± 93.51	0.551	145.81 ± 127.45	99.51 ± 82.29	0.033
ALP, units per liter	296.36 ± 127.84	358.29 ± 260.97	0.205	418.67 ± 238.94	329.01 ± 236.34	0.101	280.47 ± 123.64	355.80 ± 252.34	0.184
Albumin	3.63 ± 0.64	3.32 ± 0.44	0.003*	3.40 ± 0.51	3.39 ± 0.51	0.908	3.27 ± 0.46	3.41 ± 0.52	0.261

## Discussion

According to the current study findings, the etiological spectrum of CLD showed that hepatitis B was the most common cause, followed by idiopathic cause, GSD, Wilson disease, biliary atresia, autoimmune and progressive familial intrahepatic cholestasis, tyrosinemia type-I, galactosemia, Budd-Chiari syndrome, neonatal hepatitis, Niemann-Pick disease, and choledochal cyst, while citrin deficiency and hypothyroidism were observed in one patient each.

Irrespective of the etiology, jaundice and abdominal distention are the most common presenting features of CLD as reported by Dhole et al. [9]. The reported prevalence of metabolic disorders like Wilson disease is not uncommon, and it varies from 16% to 21% [10,11]. Dhole et al. found that 22% of their patients had Wilson disease on histopathology [9]. Recently, non-alcoholic steatohepatitis (NASH) is considered a common etiological factor for CLD among children. Tominaga et al. reported a prevalence of 2.6-12.5% in their study [12]. In Asia, hepatitis B virus (HBV) is the leading cause of pediatric CLD (74.4% of all cases) as reported by Zhang et al. [13]. Hanif et al. [10] reported that HBV is the most common cause of pediatric CLD at 24%, and the risk of chronicity is about 90% when the infection is acquired in infancy [14].

Tahir et al. [15], while studying children presenting with CLD at Fauji Foundation Hospital, Rawalpindi, reported viral hepatitis as the most common etiology with the majority of such patients suffering from hepatitis C. Arif and Thejeal [16] in another similar local study involving liver biopsy specimens reported metabolic diseases including GSD and familial intrahepatic cholestasis being the frequent etiologies of CLD in the pediatric population.

Muthuphei [17] reported biliary atresia (20.8%) and neonatal hepatitis (19.4%) as common etiologies of CLD in South African children. Another large-scale Pakistani study reported idiopathic CLD, biliary atresia, neonatal hepatitis, and Wilson disease as the common hepatobiliary disorders [18]. Moreover, treatable and preventable causes like hepatotropic viral infections are still a major problem in our setup [15-18].

The affected child may remain silent, may only have biochemical evidence of liver dysfunction, or can present with acute liver failure. Children usually have similar clinical presentations but management substantially varies depending on the cause [19].

The profile of metabolic disease-producing CLD in developing countries has not been well documented because of the lack of diagnostic facilities [20]. Evidence from western countries suggests that autoimmune hepatitis is the leading cause of CLD, and infections are among the lowest on the causative list [21]. Liver metabolic disorders have not been well documented in developing countries because of a lack of diagnostic facilities.

As per the current study findings, a significant association of hepatitis was observed with age, residence, symptomatic, total bilirubin level, direct bilirubin level, and albumin level. Whereas a significant association of GSD was observed with age, residence, and SGPT level. Furthermore, slight male predominance was reported in the current study.

The findings of the study could be highlighted in the light of the limitation that this was a single-center study and was conducted on a limited sample size. Furthermore, in the current study, certain important factors such as biochemical parameters, biopsy findings, prothrombin time, prothrombin index, bleeding time, and clotting time were not studied. Despite these limitations, this study is of importance as it has generated local study findings that would be beneficial for both patients and healthcare professionals. Further large-scale multicenter studies are recommended that can preclude the findings of this study.

## Conclusions

In our cohort, hepatitis B, idiopathic, GSD, and Wilson disease were the most common causes of CLD in pediatric patients. Males were predominantly higher. Whereas age, residence, symptomatic, total bilirubin level, direct bilirubin level, SGPT, and albumin level were the important predictor variables. Additionally, hepatomegaly was observed amongst almost half of the study participants. Identification of the spectrum of the disease will help in the early preventive measures. The present study findings would be helpful in taking the relevant preventive measures accordingly, such as some metabolic disorders can be treated with some diet modifications if diagnosed early.
